# Relationship between cardiac index and brain volume in the UK Biobank

**DOI:** 10.1016/j.cccb.2026.100560

**Published:** 2026-08-21

**Authors:** Sarah Syed, Chris Moran, Stephanie Than, Jarin Herson, Taya Collyer, Richard Beare, Velandai Srikanth

**Affiliations:** a Department of Home, Acute and Community, Alfred Health, 260 Kooyong Rd, Caulfield, Victoria, 3162, Australia; b Faculty of Medicine, Nursing and Health Sciences, Medicine Monash Health, Monash University, Melbourne, Australia; c School of Public Health and Preventive Medicine, Monash University, 553 St Kilda Road, Melbourne, 3004, Victoria, Australia; d National Centre for Healthy Ageing, PO Box 52, Frankston, Victoria, 3199, Australia; e Department of Geriatric Medicine, Western Health, 160 Gordon Street, Footscray, 3011, Australia; f Department of Medicine, Melbourne Medical School, The University of Melbourne, Melbourne, Australia; g Department of Geriatric Medicine, Peninsula Health, 24 Separation Street, Mornington, Victoria, 3931, Australia; h Peninsula Clinical School, School of Translational Medicine, Monash University, PO Box 52, Frankston, Victoria, 3199, Australia; i Developmental Imaging, Murdoch Children’s Research Institute, Melbourne, 50 Flemington Rd, Parkville, Victoria, 3052, Australia

**Keywords:** Cardiac index, Brain, Ageing, Imaging, MRI

## Abstract

**Aims:**

Although cardiac function is associated with cognitive impairment in end-stage heart disease, the links between cardiac function and brain structure remain unclear. We aimed to examine the association between cardiac index (CI) (a measure of cardiac output adjusting for body surface area) and brain volumes in mid-to later life and how these are affected by age, sex and cardiometabolic factors.

**Materials and methods:**

We utilized brain and cardiac magnetic resonance imaging (MRI) data from a population-based cohort of people in the UK Biobank (a United Kingdom population-based cohort of people > 40 years old) at mid to later life. Linear regression modelling was used to examine associations of CI with total brain volume (TBV), gray matter (GMV), white matter (WMV), white matter hyperintensity (WMHV) and total hippocampal volume (THV), and assess whether these associations were influenced by sex, age or cardiometabolic factors.

**Results:**

Complete data were available for 22,199 individuals (mean age 64 years [SD 7], 64% women). In univariable analyses, lower CI was associated with lower TBV, GMV, WMV, THV and greater WMHV (p < 0.001). In adjusted models, statistically significant CI × age interactions were observed for TBV, GMV, and THV (all p < 0.01) indicating more pronounced associations in older individuals. No CI × sex interactions were identified.

**Conclusions:**

Lower cardiac index is associated with lower brain volumes and greater vascular lesion burden in mid-to later life, with evidence of age modification for TBV, GMV and THV.

## Highlights

• Cardiac index is a measure of cardiac output adjusted for body surface area.
• Cardiac dysfunction and brain health are strongly associated through several complex mechanisms.
• Lower cardiac index is associated with lower brain volumes in multiple brain regions.
• There was evidence of age modification for some brain-volume outcomes, particularly total brain, gray matter, and hippocampal volume.

## 1 Introduction

Cardiac dysfunction and heart failure (HF) is associated with an increased risk of dementia [[1], [2], [3], [4], [5]]. A number of mechanisms are thought to be responsible for this association including disruptions to cerebral blood flow (CBF), cerebral hypoperfusion and ultimately ischemic cerebral damage [6,7]. Although previous work has reported strong associations between HF and dementia [5,8], the time of life in which HF occurs appears to be important. In the Cardiovascular Risk Factors, Aging and Dementia study, a population-based study over 25 years of follow up, authors reported that HF in late life, but not mid to later life, was associated with a two-fold increased risk of dementia [9]. If HF is causally associated with dementia risk, the results of previous work suggest that it is possible that the competing risk of death in people in mid to later life with HF reduces the ability to identify these associations. One approach to explore the potential contribution of HF to dementia risk is to examine associations between early markers of cardiac dysfunction and sensitive markers of brain health in people in mid to later life. Advances in structural imaging using magnetic resonance imaging (MRI) now allow for the characterization of both cardiac and brain health.

Cardiac MRI is widely acknowledged as a reference standard for quantification of ventricular volumes and function by accurately assessing left ventricular function and cardiac output [10]. Cardiac index (CI) is used as a measure of cardiac function that allows across-person comparisons that account for differences in cardiac output related to body size (surface area). Similarly, MRI has excellent brain tissue penetration, allowing for accurate calculation of brain structure volumes as surrogate markers of brain health [11]. We therefore aimed to explore the associations between MRI-measured cardiac function and brain volumes in a large cohort of people, and to examine how these associations are affected by sex, age and cardiometabolic factors.

## 2 Materials and methods

### 2.1 Study participants and data

We acquired data from the UK Biobank, a large-scale biomedical database and international open access-resource which was launched with the primary aim of developing a deeper understanding of diseases of middle age and later life (www.ukbiobank.ac.uk). The cohort consists of over 500,000 volunteers aged 40 to 69 years who were registered with National Health Service (NHS) and lived within traveling distance of 22 assessment centers across the United Kingdom. The initial cohort was recruited between 2006 and 2010, and the database is regularly augmented with additional data. Complete details of the recruitment process and other key UK Biobank procedures are available on www.ukbiobank.ac.uk. Informed consent was obtained from all participants. Assessments involved the collection of extensive lifestyle and health information in the form of questionnaires, physical and cognitive measures and analysis of biological samples (including blood, urine and saliva samples). In 2014, the UK Biobank commenced magnetic resonance imaging (MRI), and 100,000 participants of the original cohort were invited to undertake detailed brain and cardiac MRI. For this study, we only included data of individuals who underwent both cardiac magnetic resonance imaging and brain magnetic resonance imaging and had complete metabolic data available.

The UK Biobank approved the study application (Project ID 24954) and we obtained ethics approval from the UK Biobank Research Ethics Committee (reference 11/NW/0382). We also received ethics approval from the Monash University Human Research Ethics Committee (project ID:18734).

### 2.2 Clinical and demographic data

Demographic information and medical history were collected during each participant’s attendance for MRI. We used age at time of MRI attendance for our analysis. Variables including ethnicity, educational qualifications, and smoking and alcohol use were based on self-reported data. Participant ethnicity in the UK Biobank was derived from self-reported answers to a questionnaire, rather than genetically inferred ancestry. Socioeconomic status was classified using the Townsend deprivation index [12].

Participants were classified as *ApoE ε4* positive if they carried at least one *ApoE ε4* allele. Cardiometabolic risk factors were classified based on a combination of self-report on questionnaire, medication use and ICD-10 codes. Participants classified as having hypertension included those who had a self-reported diagnosis of hypertension, those who were taking anti-hypertensive medication and/or had ICD-10 codes for hypertension. Similarly, diabetes was classified as those participants who self-reported a diabetes diagnosis or used glucose lowering medication (including insulin). A similar approach was taken to classify patients into categories of ischemic heart disease (IHD), hypercholesterolemia and stroke. Participants who selected “prefer not to answer” or “not sure” to touchscreen questions in relation to cardiometabolic health conditions were coded as missing (<1%). We excluded those with a diagnosis of ischemic heart disease or an ICD-10 code for cardiomyopathy to reduce the potential of confounding from overt structural health disease where reductions in CI may be driven by established myocardial pathology rather than physiological variation. Participants were also excluded if they had incomplete data available for systolic and diastolic blood pressure recordings.

### 2.3 Brain MRI acquisition and processing

Brain MRI measures in the UK Biobank were obtained as part of the UK Biobank Imaging Study using a standardized multimodal protocol on Siemens Skyra 3T scanners (Siemens Skyra, software VD13, Siemens Healthcare) with 32-channel radiofrequency head coils.

Participants were scanned at dedicated UK Biobank imaging assessment centres. To minimise scanner effects, UK Biobank brain MRI data were acquired using a harmonised acquisition protocols across imaging centres, with identical Siemens Skyra 3T scanners, VD13 software, 32-channel head coils, standardised radiographer training, routine phantom and performance checks, and centralised image processing and quality control procedures.

Head size was defined using the UK Biobank T1-derived volumetric scaling factor, computed from registration of the participant’s T1 head image to standard MNI152 space after estimating the external skull surface. This scaling factor was used to normalise volumetric brain imaging-derived phenotypes for individual differences in head size. The brain MRI protocol included T1-weighted, T2-FLAIR, susceptibility-weighted, diffusion, resting-state fMRI, and task fMRI sequences. The measures that were utilized in our analysis included total brain volume (TBV), total gray matter volume (GMV), total white matter volume (WMV) and total hippocampal volume (THV) which were derived from T1 scans, and normalized for head size. The volume of total white matter hyperintensity (WMHV) was calculated using the BIANCA tool [13] applied to T1 and T2 FLAIR imaging. The complete UK Biobank MRI acquisition protocol can be found at: https://biobank.ndph.ox.ac.uk/showcase/refer.cgi?id=2367. We relied on quality control and careful calibration procedures implemented by UK Biobank to avoid site effects as suggested by the analysis protocol (https://biobank.ndph.ox.ac.uk/showcase/refer.cgi?id=1977). This approach is supported by recently published work reporting high reliability between sites [14].

### 2.4 Cardiac MRI acquisition and processing

Participants underwent cardiac MRI on a 1.5T wide bore scanner (MAGNETOM Aera, Syngo Platform VD13A, Siemens Healthcare) https://biobank.ndph.ox.ac.uk/showcase/refer.cgi?id=349. Automated image analysis was performed by software provided by the scanner to determine end-diastolic and end-systolic volumes to calculate cardiac index, which is calculated as cardiac output divided by body surface area. This approach is considered superior to using cardiac output alone as it effectively corrects for the patient’s body size, thus creating a normalized value for cardiac function.

Tukey’s method, a validated and effective method to exclude outliers from normally-distributed data [15], was used to exclude significant cardiac index outlier values to reduce the influence of established myocardial pathology rather than physiological variation. Values were considered outliers if they were >1.5 times the interquartile range from the quartile, i.e., either below Q1 – 1.5x IQR or above Q3 + 1.5x IQR. This was applied to all values of left ventricular end-systolic volume (LVESV), left ventricular end-diastolic volume (LVEDV) and stroke volume (SV).

### 2.5 Statistical analysis

Simple summary statistics were used to describe the sample. WMHV was positively skewed, therefore it was natural-log transformed before regression analyses. We used multivariable linear regression to study associations between MRI-calculated CI with TBV, WMV, GMV, WMHV and HV (whereby the beta-coefficient represents the estimated change in brain volume in millilitres (mL) associated with each 1L/min/m^2^ increase in cardiac index). To aid interpretation, we also present the beta coefficient for the univariable association between age and brain volume (whereby the beta-coefficient represents the estimated change in brain volume (mL) associated with each 1-year increase in age). Our previous work has reported the presence of an age × sex interaction on the brain volumes used in the UK Biobank [[16], [17], [18], [19]]. We therefore analysed for the presence of an age × sex interaction, and whether there were also CI × age and CI × sex interaction terms. We then built on these minimally adjusted models by including a fully adjusted model to explore whether CI shared an association with brain volume independent of potentially intermediary pathways. This fully adjusted model included modifiable and non-modifiable cardiovascular risk factors, demographic factors and socioeconomic factors, namely ethnicity, *ApoE ε4* status, Townsend Deprivation Index, systolic blood pressure, diastolic blood pressure, diabetes, hypertension, stroke, hypercholesterolemia, Body Mass Index, alcohol and smoking status. To ensure model stability, multi-collinearity among mean-centred covariates was assessed using Variance Inflation Factors (VIF). All final model covariates demonstrated acceptable independence with VIF values below 5 (Supplementary Table 1). Interactions that were identified were further explored in two ways. First, interactions were visually examined using scatter plots. Interactions involving age as a continuous variable were plotted with age categorized into tertiles of <58 years old, between 58 and 68 years old, and >68 years old, based on the distribution of age in the sample. Second, between-slope differences were statistically evaluated using Wald tests with the <58 years group specified as the reference category.

To reduce the risk of false positives from multiple comparisons, we used Bonferroni correction (0.05/5 = p < 0.01) across each of the five brain volumes outcomes. All statistical analysis was performed using RStudio (version 1.4.1717) in R (version 4.1.1).

## 3 Results

### 3.1 Sample characteristics

Of 27,157 individuals, 4958 were excluded due to extreme outlier values in LVEDV, LVESV and SV on cardiac MRI, leaving an analytical cohort of 22,199. Table 1 describes the characteristics of the participants, with further stratification into tertiles of age. The mean age of the sample was 64.1 years (SD = 7.4). Women made up 64% (n = 14,198) of participants. Almost 50% of participants had a tertiary degree. >90% of participants self-identified as British. The prevalence of hypertension was 32% and diabetes was 5%. Over one-third-of participants (36%) reported having ever smoked. A total of 5948 participants (27%) carried at least one *ApoE ε4* allele. The mean systolic and diastolic blood pressure of participants was 140 mmHg (SD = 20) and 78 mmHg (SD = 11) respectively. The mean BMI of the sample was 26.1 kg/m^2^ (SD = 4.3). The mean CI was 2.5 L/min/m^2^ (SD = 0.5).

**Table 1:** Participant characteristics.

	Whole Group	<58 years old	58–68 years old	>68 years old
	Mean ± SD *or* median (IQR) *or* n (%)	Mean ± SD *or* median (IQR) *or* n (%)	Mean ± SD *or* median (IQR) *or* n (%)	Mean ± SD *or* median (IQR) *or* n (%)
n	n = 22,199	n = 5291	n = 9423	n = 7485
Age (years)	64.1 ± 7.4	53.9 ± 2.6	63.3 ± 2.9	72.1 ± 3.0
Women	14,198 (64%)	3753 (71%)	6241 (66%)	4204 (56%)
Tertiary degree	10,840 (49%)	2810 (53%)	4710 (50%)	3320 (44%)
Townsend Deprivation Index	−2.6 (IQR 3.3)	−2.3 (IQR 3.3)	−2.6 (IQR 3.3)	−2.8 (IQR 3.0)
Self-reported ethnicity				
British	20,234 (91%)	4646 (89%)	8582 (91%)	7006 (94%)
Other White	653 (2.9%)	187 (3.5%)	277 (2.9%)	189 (2.5%)
Irish	523 (2.4%)	141 (2.7%)	253 (2.7%)	129 (1.7%)
Indian	170 (0.8%)	67 (1.3%)	57 (0.6%)	46 (0.6%)
Other ethnic group	122 (0.5%)	39 (0.7%)	48 (0.5%)	35 (0.5%)
Hypertension	7019 (32%)	1087 (20%)	2841 (30%)	3091 (41%)
Diabetes mellitus	1081 (5%)	177 (3%)	443 (5%)	461 (6%)
Hypercholesterolemia	4508 (20%)	364 (7%)	1628 (17%)	2516 (34%)
Stroke	677 (3%)	143 (2%)	305 (3%)	229 (3%)
Ever smoked	7948 (36%)	1610 (30%)	3295 (35%)	3029 (40%)
Ever drunk alcohol	21,318 (96%)	5073 (96%)	9100 (97%)	7145 (95%)
Apolipoprotein E (*APO*ε4) carrier	5948 (27%)	1469 (28%)	2601 (27%)	1861 (25%)
Systolic blood pressure (mmHg)	140 ± 20	130 ± 17	139 ± 19	147 ± 20
Diastolic blood pressure (mmHg)	78 ± 11	78 ± 11	79 ± 10	78 ± 11
Body mass index (kg/m^2^)	26.1 ± 4.3	26.2 ± 4.6	26.1 ± 4.3	26.0 ± 3.9
Cardiac index (L/min/ m^2^)	2.5 ± 0.5	2.6 ± 0.5	2.5 ± 0.5	2.3 ± 0.5
Total brain volume (mL)	1498 ± 73	1555 ± 62	1505 ± 62	1451 ± 61
Gray matter volume (mL)	797 ± 47	837 ± 39	801 ± 38	764 ± 40
White matter volume (mL)	702 ± 41	718 ± 37	704 ± 39	687 ± 41
Volume of white matter hyperintensity (mL)	4.7 ± 6.2	2.1 ± 2.8	3.9 ± 4.7	7.5 ± 8.0
Total hippocampal volume (mL)	10 ± 1.1	10.4 ± 1.0	10.1 ± 1.1	9.6 ± 1.1

### 3.2 CI, age, sex and brain volumes

Table 2 describes the associations between CI, TBV, GMV, WMV, log-transformed WMHV and THV across 3 different models: 1) univariable, 2) adjusted for age × sex interaction and 3) fully adjusted for sociodemographic and cardiometabolic variables. Supplementary Table 2 presents more detailed description of all 5 models examined: 1) univariable, 2) adjusted for age × sex interaction, 3) Model 2 plus CI × sex interaction term, 4) Model 2 plus CI × age interaction term and 5) Model 4 fully adjusted for sociodemographic and cardiometabolic variables.

**Table 2:** Associations and interactions between cardiac index (CI), age and sex on brain volumes.

	Model 1 β (95%CI) p-value	Model 2 β (95%CI) p-value	Model 3 β (95%CI) p-value
**Total brain volume** (mL)			
CI	18.63 (16.65 to 20.60) <2 × 10^−16^	2.66 (1.02 to 4.30) 1.48×10^−3^	−20.44 (−34.97 to −5.91) 0.006
Age × Sex	N/A	0.77 (0.55 to 0.99) 7.4 × 10^−12^	0.80 (0.57 to 1.024) 4.46×10^−12^
**Gray matter volume** (mL)			
CI	10.77 (9.48 to 12.06) <2 × 10^−16^	−0.79 (−1.77 to 0.20) 0.12	−14.43 (−23.09 to −5.77) 0.001
Age × Sex	N/A	0.45 (0.32 to 0.59) 1.8 × 10^−11^	0.47 (0.33 to 0.60) 9.1 × 10^−12^
**White matter volume** (mL)			
CI	7.85 (6.75 to 8.96) <2 × 10^−16^	3.45 (2.38 to 4.51) 2.1 × 10^−10^	−6.01 (−15.45 to 3.44) 0.21
Age × Sex	N/A	0.32 (0.17 to 0.46) 1.43×10^−5^	0.33 (0.18 to 0.48) 1.1 × 10^−5^
**Log-transformed white matter hyperintensity volume**			
CI	−0.17 (−0.19 to −0.14) <2 × 10^−16^	0.02 (0.0002 to 0.048) 0.048	0.10 (−0.11 to 0.31) 0.34
Age × Sex	N/A	−0.01 (−0.013 to −0.006) 1.56×10^−9^	−0.008 (−0.01 to −0.005) 3.62×10^−7^
**Total hippocampal volume** (mL)			
CI	0.17 (0.14 to 0.20) <2 × 10^−16^	0.03 (0.01 to 0.06) 0.03	−0.31 (−0.56 to −0.05) 0.02
Age × Sex	N/A	−0.17 (−0.02 to −0.1) <2 × 10^−16^	−0.02 (−0.02 to −0.01) 6.06×10^−16^

**Key**: Model 1: univariable; Model 2: Model 1 + age × sex interaction term; Model 3: Model 2 + ethnicity, ApoE ε4 status, Townsend Deprivation Index, systolic blood pressure, diastolic blood pressure, diabetes, hypertension, stroke, hypercholesterolemia, Body Mass Index, alcohol and smoking status.

#### 3.2.1 Total brain volume

Lower CI was associated with lower TBV (Beta coefficient [β] = 18.63 mL, 95% confidence interval [CI] 16.65 to 20.60, p < 0.0001), i.e., a 1 L/min/m² decrease in CI associated with 18.63 mL decrease in TBV (Table 2, Model 1). To aid interpretation, the univariable association of age with TBV was −5.66 mL (95% CI −5.77 to −5.56). The addition of the known statistically significant age × sex interaction term (β = 0.77 mL, 95% CI 0.55 to 0.99, p < 0.001) resulted in a weakening of the association between CI and TBV (β = 2.66 mL, 95% CI 1.02 to 4.30, p = 1.48×10^−3^, Model 2). Although we did not find an interaction between CI and sex (p = 0.65), an interaction between CI and age was found (p = 9.2 × 10^−4^), which persisted when fully adjusted for sociodemographic and cardiometabolic variables (p = 0.003).

**Fig. 1:**
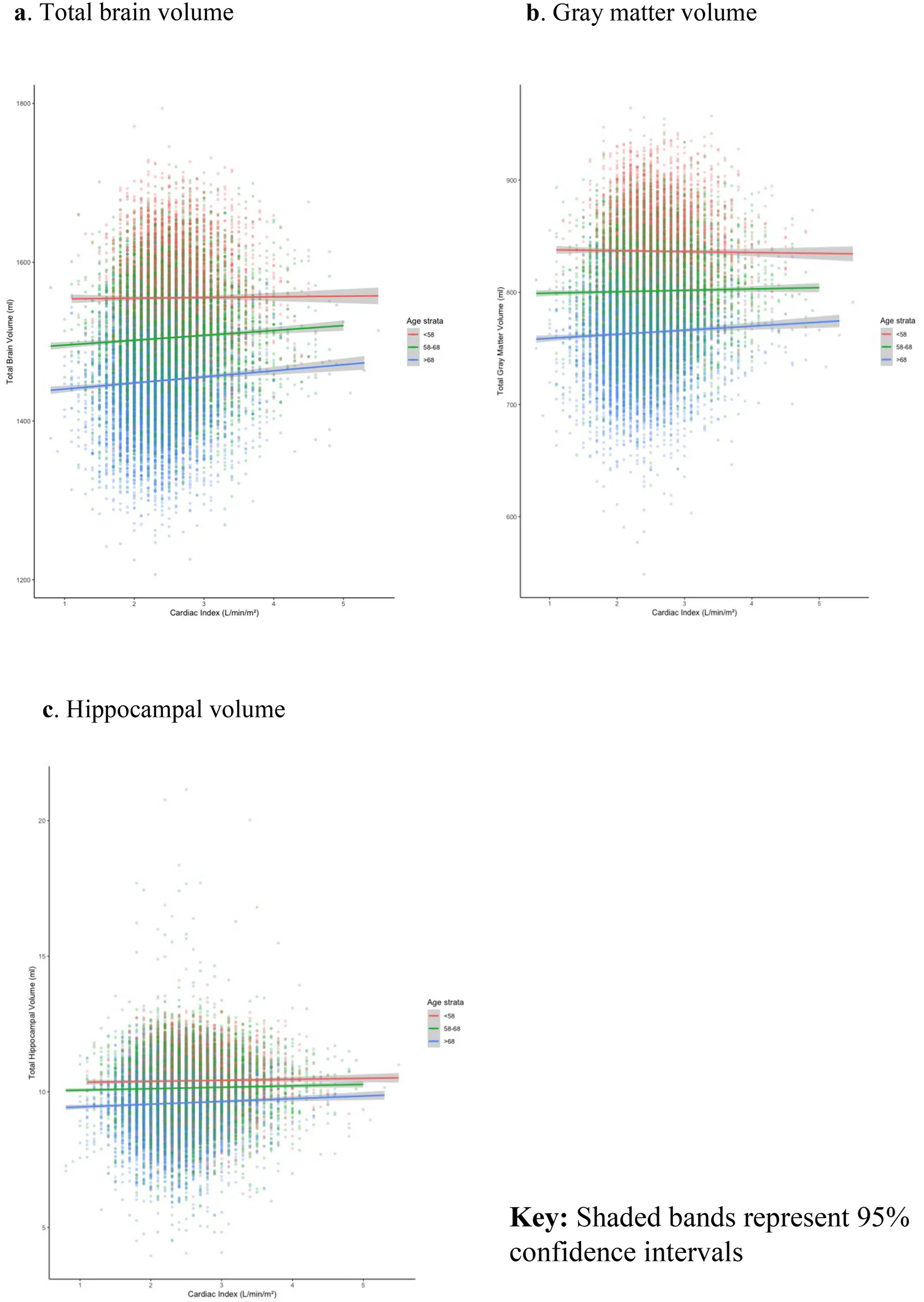
Association between cardiac index and brain volumes stratified by age.

Fig. 1a presents predicted TBV plotted against CI stratified by age (<58 years old, 58–68 years old, >68 years old). Relative to those <58 years of age, the association between CI and TBV was more positive in those over 68 years (difference in slope from <58 years of age = 6.74 mL, p = 0.004) and those aged 58–68 years (difference in slope from <58 years of age = 5.69 mL, p = 0.009) (Table 3). There was a statistically significant association between CI and TBV in the 58–68 years (β = 6.15 mL, 95% CI 3.57 to 8.73, p = 3.05×10^−6^) and >68 strata (β = 7.67 mL, 95% CI 4.74 to 10.59, p = 2.91×10^−7^) but not in those <58 years of age (β = 0.84 mL, 95% CI −2.68 to 4.37, p = 0.64) (Supplementary Table 3).

**Table 3:** Association between cardiac index and brain volumes in age strata relative to those under 58 years of age.

	Total brain volume Slope difference from those <58 years (95%CI) p-value	Gray matter volume Slope difference from those <58 years (95%CI) p-value	White matter volume Slope difference from those <58 years (95%CI) p-value	Log-transformed white matter hyperintensity volume Slope difference from those <58 years (95%CI) p-value	Total hippocampal volume Slope difference from those <58 years (95%CI) p-value
Age <58	Reference	Reference	Reference	Reference	Reference
Age 58–68	5.69 (1.37 to 10.00) 0.009	2.43 (−0.19 to 5.05) 0.07	3.26 (0.52 to 5.99) 0.02	0.11 (−0.31 to 0.52) 0.61	19.55 (−55.56 to 94.65) 0.61
Age >68	6.74 (2.19 to 11.29) 0.004	3.82 (1.05 to 6.58) 0.007	2.92 (0.04 to 5.81) 0.04	−1.11 (−0.54 to 0.33) 0.64	35.89 (−43.34 to 115.13) 0.37

#### 3.2.2 Gray matter volume

Lower CI was associated with lower GMV (β= 10.77 mL, 95% CI 9.48 to 12.06, p < 0.001) (Table 2). To aid interpretation, the univariable association of age with GMV was −3.96 mL (95% CI −4.02 to −3.89). The addition of the age × sex interaction resulted in the association between CI and GMV no longer being statistically significant (p = 0.12, Model 2). There was no interaction between CI and sex (p = 0.73). There was an interaction between CI and age (β = 0.22, 95% CI 0.09 to 0.36, p = 8.9 × 10^−4^), and this interaction persisted when fully adjusted for sociodemographic and cardiometabolic variables (p = 0.004).

Fig. 1b presents predicted GMV plotted against CI stratified by age (<58 years old, 58–68 years old, >68 years old). Relative to those <58 years of age, the association between CI and GMV was visually more positive in people >68 years (difference in slope from <58 years of age =3.82, p = 0.007) (Table 3). There was not a statistically significant difference between the slopes for those <58 and those between 58–68 years of age (p = 0.07). There was a statistically significant association between CI and GMV in those >68 strata (β = 3.61 mL, 95% CI 1.71 to 5.49, p = 0.0002) but not those <58 years of age (p = 0.49) or between 58–68 years of age (p = 0.14) (Supplementary Table 3).

#### 3.2.3 White matter volume

Lower CI was associated with lower white matter volume (β= 7.85 mL, 95% CI 6.75 to 8.96, p ≤ 2 × 10^−16^) (Table 2, Model 1). To aid interpretation, the univariable association of age with WMV was −1.70 mL (95% CI −1.77 to −1.63). The addition of the known age × sex interaction weakened the association between CI and WMV (β =3.45 mL, 95% CI 2.38 to 4.51, p = 2.1 × 10^−10^). We did not find a statistically significant interaction between CI and sex (p = 0.31) or CI and age (p = 0.04) on WMV.

#### 3.2.4 White matter hyperintensity volume

Lower CI was associated with greater log-transformed WMHV (β = −0.17, 95% CI −0.19 to −0.14, p < 2 × 10^−16^). To aid interpretation, the univariable association of age with log-transformed WMHV was 0.069 (95% CI 0.067 to 0.071). The addition of the age × sex interaction resulted in the association between CI and log-transformed WMHV no longer being statistically significant (p = 0.048, Table 2).

#### 3.2.5 Total hippocampal volume

Lower CI was associated with lower THV (β = 0.17 mL, 95% CI 0.14 to 0.20, p < 2 × 10^−16^) (Table 2). To aid interpretation, the univariable association of age with THV was −0.05 mL (95% CI −0.048 to −0.045). The addition of the age × sex interaction term weakened the association between CI and THV (β=0.03 mL, 95% CI 0.01 to 0.06, p = 0.03) (Model 2). There was no interaction between CI and Sex (p = 0.08). There was an interaction between CI and age (β=0.005, 95% CI 0.001 to 0.009, p = 0.01). These interactions were minimally changed in fully adjusted models. When this interaction was examined using age strata rather than age as a continuous variable, the CI × age strata interaction was no longer statistically significant (p = 0.79).

Fig. 1c presents predicted THV plotted against CI stratified by age. Relative to those <58 years of age, we found the association between CI and THV was similar in both those aged 58–68 years (difference in slope from <58 years of age=16.55, p = 0.61) as well as those >68 years (difference in slope from <58 years of age=35.89, p = 0.37). There was a statistically significant association between CI and THV in those >68 strata (β = 0.10 mL, 95% CI 0.04 to 0.15, p = 0.0003) but not those <58 years of age (p = 0.23) or between 58–68 years of age (p = 0.02) (Supplementary Table 3).

#### 3.2.6 Sensitivity analyses

The inclusion of those with significant cardiac index outlier values resulted in minimal change to the characteristics of the cohort (Supplementary Table 3) or the associations between CI and the brain volumes reported above (Supplementary Table 5).

## 4 Discussion

In this large, community-based sample of over 22,000 participants, we found that lower cardiac index was associated with poorer total brain, gray matter, white matter, white matter hyperintensity and hippocampal volume and that there was evidence of age modification for total brain, gray matter, and hippocampal volume. In the case of total brain and gray matter volume, the association between lower CI and poorer brain volumes was least evident in the youngest tertile. These results support current work exploring the links between cardiovascular and brain health [[20], [21], [22], [23], [24]] and suggest that this relationship may vary by age.

Our results are consistent with the only other study to our knowledge which examined associations between cardiac index and brain structure [25]. That study, published in 2010, reported that greater CI was associated with greater total brain volume and lower lateral ventricular volume in 1504 participants in the Framingham Offspring Cohort [25]. The authors also reported an interaction between CI and age, and CI and sex, on total brain volume such that the association between CI and total brain volume was present only in individuals under 60 years of age, and only in men. In contrast, our analyses identified subtle CI-age interactions with total brain and gray matter volume across all age groups and in both sexes. One possibility for this difference is the greater statistical power available in our study, with a sample size over 10 times larger than the Framingham cohort, with 15,332 participants over the age of 60.

The mechanisms that tie CI and brain health are yet to be fully understood. One hypothesis that has been suggested includes factors related to cerebral perfusion [4,7,22,[26], [27], [28], [29], [30], [31]]. The closest human model that is currently available to examine the influence of reduced cardiac output on brain health is chronic HF. Severe HF is characterised by reduced cardiac output and reductions in cerebral blood flow by as much as 30% [26,[32], [33], [34]]. It is challenging to separate the role of potential confounders from the direct effects of reduced cardiac output on brain health, as HF and cognitive impairment both increase in prevalence with age and share common risk factors such as hypertension, atrial fibrillation and coronary artery disease [22,35,36]. Recent work has shown heart failure to be associated with reduced carotid blood flow and subsequent cognitive dysfunction (executive and attention changes) as well as a greater dementia risk [23,28,32,35,[37], [38], [39], [40]]. Systemic changes in sympathetic tone associated with HF may also cause chronic cerebral vasoconstriction and therefore contribute to impaired cerebral blood flow autoregulation [33,41]. Dysautoregulation of cerebral blood flow may lead to direct neuronal damage through ischemia, and also potentially to impaired clearance of amyloid and tau from cerebral spinal fluid [42,43]. These adverse changes may result in more rapid deterioration in brain health through the direct toxicity of soluble oligomers as well as damage to infrastructure supporting neurones [28,43,44].

This study has a number of strengths, in particular the very large population-based cohort with extensive demographic and socio-economic data available. This allowed us to perform detailed analysis with substantial statistical power to explore complex relationships. The use of precise cardiac and brain imaging data and the stringent quality control of the UK Biobank database increases the confidence in our results. A limitation of this study is the inherent selection bias due to the nature of volunteer participants who are usually more health-literate and less ethnically diverse than the population from which they are recruited. This may have implications for the overall generalisability of the results. The exclusion of individuals with overt cardiac disease to focus on physiological variation also introduces important nuances as many had low CI values. This suggests that our sample is not limited to those with good cardiovascular health but likely includes a spectrum encompassing subclinical variations in cardiac function, age-related physiological decline, and potentially undiagnosed, incipient cardiac disease. As such, the observed associations between lower CI and reduced brain volumes may be driven by early, asymptomatic hemodynamic insufficiency rather than purely healthy physiological variance. Lastly, the cross-sectional design of the study increased the likelihood of confounding and limits inferences of causality.

## 5 Conclusion

Poorer CI was associated with poorer total brain, gray matter, white matter, white matter hyperintensity and hippocampal volume. There was evidence of age modification for some brain-volume outcomes, particularly total brain, gray matter, and hippocampal volume. Future work is required to understand longitudinal associations, and potential mechanisms by which cardiac index may contribute to brain health in mid to later life.

## CRediT authorship contribution statement

**Sarah Syed:** Writing – review & editing, Writing – original draft, Methodology, Investigation, Formal analysis, Conceptualization. **Chris Moran:** Writing – review & editing, Supervision, Formal analysis, Conceptualization. **Stephanie Than:** Supervision, Methodology, Data curation, Conceptualization. **Jarin Herson:** Methodology. **Taya Collyer:** Data curation. **Richard Beare:** Validation, Software, Resources, Formal analysis, Conceptualization. **Velandai Srikanth:** Writing – review & editing, Supervision, Conceptualization.

## Declaration of competing interest

The authors declare that they have no known competing financial interests or personal relationships that could have appeared to influence the work reported in this paper.

## Appendix Supplementary materials

Image, application 1

## References

[1] Wolters F.J. (2018). Coronary heart disease, heart failure, and the risk of dementia: a systematic review and meta-analysis. Alzheimers Dement..

[2] Jefferson A.L. (2015). Low cardiac index is associated with incident dementia and Alzheimer disease: the Framingham heart study. Circulation.

[3] Gardener H. (2015). Brain health and shared risk factors for dementia and stroke. Nat. Rev. Neurol..

[4] Hoth K.F. (2008). Cardiac dysfunction and cognition in older adults with heart failure. Cogn. Behav. Neurol..

[5] Qiu C., Fratiglioni L. (2015). A major role for cardiovascular burden in age-related cognitive decline. Nat. Rev. Cardiol..

[6] Choi B.-R. (2006). Factors associated with decreased cerebral blood flow in congestive heart failure secondary to idiopathic dilated cardiomyopathy. Am. J. Cardiol..

[7] Alosco M.L. (2013). The adverse effects of reduced cerebral perfusion on cognition and brain structure in older adults with cardiovascular disease. Brain Behav..

[8] Gure T.R. (2012). Prevalence of cognitive impairment in older adults with heart failure. J. Am. Geriatr. Soc..

[9] Rusanen M. (2014). Heart diseases and long-term risk of dementia and Alzheimer's disease: a population-based CAIDE study. J. Alzheimers Dis..

[10] Rajiah P.S., Francois C.J., Leiner T. (2023). Cardiac MRI: state of the art. Radiology.

[11] MacDonald M.E., Pike G.B. (2021). MRI of healthy brain aging: a review. NMR Biomed..

[12] Townsend P., Phillimore P. (2023). Beattie, health and deprivation: inequality and the north. Taylor & Francis.

[13] Griffanti L. (2016). BIANCA (Brain Intensity AbNormality Classification Algorithm): a new tool for automated segmentation of white matter hyperintensities. Neuroimage.

[14] Duff E. (2022). Reliability of multi-site UK Biobank MRI brain phenotypes for the assessment of neuropsychiatric complications of SARS-CoV-2 infection: the COVID-CNS travelling heads study. PLoS One.

[15] Seo S. (2006).

[16] Than S. (2021). Interactions between age, sex, menopause, and brain structure at midlife: a UK biobank study. J. Clin. Endocrinol. Metab..

[17] Moran C. (2024). Interactions between age, sex and visceral adipose tissue on brain ageing. Diabetes Obes. Metab..

[18] Lu A. (2024). Interactions between muscle volume and body mass index on brain structure in the UK Biobank. Front. Dement..

[19] La Hood A. (2025). Associations between physical activity and brain structure in a large community cohort. Sci. Rep..

[20] Tan C.H., Tan J.J.X. (2024). Associations of cardiac function and arterial stiffness with cerebrovascular disease. Int. J. Cardiol..

[21] Vishwanath S. (2023). Cardiovascular disease risk scores and incident dementia and cognitive decline in older men and women. Gerontology (Basel).

[22] Ye S., Huynh Q., Potter E.L. (2022). Cognitive dysfunction in heart failure: pathophysiology and implications for patient management. Curr. Heart Fail. Rep..

[23] Faulkner K.M. (2022). Factors associated with cognitive impairment in heart failure with preserved ejection fraction. J. Cardiovasc. Nurs..

[24] Giacona J.M. (2024). Associations between cardiac function and brain health in diverse middle-aged adults: the Dallas heart study-2. JACC: Adv..

[25] Jefferson A.L. (2010). Cardiac index is associated with brain aging: the Framingham heart study. Circulation.

[26] Ovsenik A., Podbregar M., Fabjan A. (2021). Cerebral blood flow impairment and cognitive decline in heart failure. Brain Behav..

[27] Potter E.L. (2021). Associations of subclinical heart failure and atrial fibrillation with mild cognitive impairment: a cross-sectional study in a subclinical heart failure screening programme. BMJ Open.

[28] Kure C.E.P. (2016). Relationships among cognitive function and cerebral blood flow, oxidative stress, and inflammation in older heart failure patients. J. Card. Fail..

[29] Austin B.P. (2011). Effects of hypoperfusion in Alzheimer's disease. J. Alzheimer's Dis..

[30] Dardiotis E. (2012). Cognitive impairment in heart failure. Cardiol. Res. Pract..

[31] Jefferson A.L. (2007). Systemic hypoperfusion is associated with executive dysfunction in geriatric cardiac patients. Neurobiol. Aging.

[32] Ampadu J., Morley J.E. (2015). Heart failure and cognitive dysfunction. Int. J. Cardiol..

[33] Castle-Kirszbaum M. (2022). Cardiac output and cerebral blood flow: a systematic review of cardio-cerebral coupling. J. Neurosurg. Anesthesiol..

[34] Gruhn N. (2001). Cerebral blood flow in patients with chronic heart failure before and after heart transplantation. Stroke.

[35] Cannon J.A. (2017). Cognitive impairment and heart failure: systematic review and meta-analysis. J. Card. Fail..

[36] van Nieuwkerk A.C. (2023). Cognitive impairment in patients with cardiac disease: implications for clinical practice. Stroke.

[37] Almeida O.P. (2012). Cognitive and brain changes associated with ischaemic heart disease and heart failure. Eur. Heart J..

[38] Bown C.W. (2020). Lower cardiac output relates to longitudinal cognitive decline in aging adults. Front. Psychol..

[39] Cheng H.-M. (2022). Education level may modify the association between cardiac index and cognitive function among elders with normal ejection function. Front. Cardiovasc. Med..

[40] de Bruijn R.F.A.G. (2015). Subclinical cardiac dysfunction increases the risk of stroke and dementia: the Rotterdam Study. Neurology.

[41] Mene-Afejuku T.O. (2019). Heart failure and cognitive impairment: clinical relevance and therapeutic considerations. Curr. Cardiol. Rev..

[42] Zheng Y.-M. (2021). Left ventricular ejection fraction and cerebrospinal fluid biomarkers of Alzheimer's Disease pathology in cognitively normal older adults: the CABLE study. J. Alzheimer's Dis..

[43] Kresge H.A. (2020). Lower left ventricular ejection fraction relates to cerebrospinal fluid biomarker evidence of neurodegeneration in older adults. J. Alzheimer's Dis..

[44] Mueller K. (2020). Brain damage with heart failure: cardiac biomarker alterations and gray matter decline. Circ. Res..

