# Supplementary material for: Relationship between cardiac index and brain volume in the UK Biobank
Source: Cereb Circ Cogn Behav. 2026 Aug 21;11:100560. doi: 10.1016/j.cccb.2026.100560 (PMC13544297; doi:10.1016/j.cccb.2026.100560)
Supplement: Supplementary file 1 [file mmc1.docx]

**Supplementary Table 1** Variance inflation factors of covariables used in fully adjusted regression models

| **Variable** | **Variance Inflation Factor** |
| --- | --- |
| Age | 2.03 |
| Sex | 1.10 |
| Cardiac Index | 1.08 |
| Age x CI | 1.00 |
| Age x Sex | 1.64 |
| Townsend Deprivation Index | 1.03 |
| British ethnicity | 1.04 |
| Hypertension | 1.18 |
| Diabetes mellitus | 1.12 |
| Hypercholesterolemia | 1.30 |
| Stroke | 1.02 |
| Ever smoked | 1.04 |
| Ever drunk alcohol | 1.03 |
| Apolipoprotein E (APOε4) carrier | 1.00 |
| Systolic blood pressure | 2.03 |
| Diastolic blood pressure | 1.85 |
| Body Mass Index | 1.17 |

**Supplementary Table 2** Detailed associations and interactions between cardiac index (CI), age and sex on brain volume

|  | **Model 1**  β  (95%CI)  p-value | **Model 2**  β  (95%CI)  p-value | **Model 3**  β  (95%CI)  p-value | **Model 4**  β  (95%CI)  p-value | **Model 5**  β  (95%CI)  p-value |
| --- | --- | --- | --- | --- | --- |
| **Total brain volume** (mL) | | | | | |
| CI | 18.63  (16.65 to 20.60)  <2×10^-16^ | 2.66  (1.02 to 4.30)  1.48×10^-3^ | 2.94  (0.90 to 4.97)  0.004 | -21.11  (-35.25 to -6.96)  0.003 | -20.44  (-34.97 to -5.91)  0.006 |
| Age × Sex |  | 0.77  (0.55 to 0.99)  7.4×10^-12^ | 0.75  (0.54 to 0.98)  2.91×10^-11^ | 0.79  (0.56 to 1.01)  2.19×10^-12^ | 0.80  (0.57 to 1.024)  4.46×10^-12^ |
| CI × Sex |  |  | -0.79  (-4.23 to 2.64)  0.65 |  |  |
| CI × Age |  |  |  | 0.37  (0.15 to 0.59)  9.2×10^-4^ | 0.35  (0.12 to 0.57)  0.003 |
| **Gray matter volume** (mL) | | | | | |
| CI | 10.77  (9.48 to 12.06)  <2×10^-16^ | -0.79  (-1.77 to 0.20)  0.12 | -0.91  (-2.14 to 0.31)  0.144 | -15.12  (-23.6 to -6.6)  0.00049 | -14.43  (-23.09 to -5.77)  0.001 |
| Age × Sex |  | 0.45  (0.32 to 0.59)  1.8×10^-11^ | 0.45  (0.32 to 0.59)  2.56×10^-11^ | 0.47  (0.33 to 0.60)  5.47×10^-12^ | 0.47  (0.33 to 0.60)  9.1×10^-12^ |
| CI × Sex |  |  | 0.36  (-1.70 to 2.43)  0.73 |  |  |
| CI × Age |  |  |  | 0.22  (0.09 to 0.36)  8.9×10^-4^ | 0.20  (0.06 to 0.33)  0.004 |
| **White matter volume** (mL) | | | | | |
| CI | 7.85  (6.75 to 8.96)  <2×10^-16^ | 3.45  (2.38 to 4.51)  2.1×10^-10^ | 3.85  (2.53 to 5.17)  1.08×10^-8^ | -5.99  (-15.15 to 3.175)  0.20 | -6.01  (-15.45 to 3.44)  0.21 |
| Age × Sex |  | 0.32  (0.17 to 0.46)  1.43×10^-5^ | 0.30  (0.16 to 0.44)  4.53×10^-5^ | 0.32  (0.18 to 0.47)  8.85×10^-6^ | 0.33  (0.18 to 0.48)  1.1×10^-5^ |
| CI × Sex |  |  | -1.15  (-3.38 to 1.07)  0.31 |  |  |
| CI × Age |  |  |  | 0.14  (0.005 to 0.29)  0.04 | 0.15  (0.003 to 0.30)  0.04 |
| **Log-transformed white matter hyperintensity volume** | | | | | |
| CI | -0.17  (-0.19 to -0.14)  <2×10^-16^ | 0.02  (0.002 to 0.004)  0.048 | 0.02  (-0.008 to 0.05)  1.16 | 0.08  (-0.12 to 0.28)  0.43 | 0.01  (-0.11 to 0.31)  0.34 |
| Age × Sex |  | -0.01  (-0.013 to -0.006)  1.56×10^-9^ | -0.009  (-0.013 to -0.007)  4.17×10^-9^ | -0.01  (-0.004 to -0.002)  1.36×10^-9^ | -0.008  (-0.01 to -0.005)  3.62×10^-7^ |
| CI × Sex |  |  | 0.007  (-0.04 to 0.06)  0.75 |  |  |
| CI × Age |  |  |  | -0.001  (-0.004 to 0.02)  0.56 | -0.001  (-0.004 to 0.001)  0.39 |
| **Total hippocampal volume** (mL) | | | | | |
| CI | 0.17  (0.14 to 0.20)  <2×10^-16^ | 0.03  (0.01 to 0.06)  0.03 | 0.012  (-0.02 to 0.04)  0.51 | -0.28  (-0.54 to -0.03)  0.027 | -0.31  (-0.56 to -0.05)  0.02 |
| Age × Sex |  | -0.17  (-0.02 to -0.1)  <2×10^-16^ | -0.02  (-0.02 to -0.01)  <2×10^-16^ | 0.005  (0.001 to 0.009)  0.01 | -0.02  ( -0.02 to -0.01)  6.06×10^-16^ |
| CI × Sex |  |  | 0.05  (-0.006 to 0.11)  0.08 |  |  |
| CI × Age |  |  |  | 0.005  (0.001 to 0.009)  0.01 | 0.005  (0.001 to 0.009)  0.01 |

**Key:** Model 1: univariable; Model 2: Model 1 + age × sex interaction term; Model 3: Model 2 + CI × sex interaction term; Model 4: Model 2 + CI × age interaction term; Model 5 Model 4 + ethnicity, ApoE ε4 status, Townsend Deprivation Index, systolic blood pressure, diastolic blood pressure, diabetes, hypertension, stroke, hypercholesterolemia, Body Mass Index, alcohol and smoking status

**Supplementary Table 3** Association between cardiac index and each of the brain volumes stratified by age group

|  | **<58 years old**  β (95% CI)  p-value | **58-68 years old**  β (95% CI)  p-value | **>68 years old**  β (95% CI)  p-value |
| --- | --- | --- | --- |
| **Total brain volume** (mL) | | | |
| Cardiac Index | 0.84  (-2.68 to 4.37)  0.64 | 6.15  (3.57 to 8.73)  3.05×10^-6^ | 7.67  (4.74 to 10.59)  2.91×10^-7^ |
| **Gray matter volume** (mL) | | | |
| Cardiac Index | -0.80  (-3.05 to 1.45)  0.487 | 1.21  (-0.41 to 2.83)  0.143 | 3.61  (1.71 to 5.49)  0.0002 |
| **White matter volume** (mL) | | | |
| Cardiac Index | 1.64  (-0.48 to 3.76)  0.13 | 4.94  (3.34 to 6.54)  1.61×10^-9^ | 4.06  (2.11 to 6.01)  4.50 ×10^-5^ |
| **Log-transformed white matter hyperintensity volume** | | | |
| Cardiac Index | 0.019  (-0.009 to 0.048)  0.19 | 0.006  (-0.01 to 0.02)  0.47 | -0.005  (-0.03 to 0.02)  0.70 |
| **Total hippocampal volume** (mL) | | | |
| Cardiac Index | 0.03  (-0.02 to 0.09)  0.226 | 0.05  (0.008 to 0.09)  0.02 | 0.10  (0.04 to 0.15)  0.0003 |

**Supplementary Table 4** Participant characteristics prior to the exclusion of those with outlier values in LVEDV, LVESV and SV.

|  | **Whole Group** |
| --- | --- |
|  | Mean ± SD *or* median (IQR) *or* n (%) |
| n | n=27,157 |
| Age (years) | 63.7 ± 7.5 |
| Women | 14837 (55%) |
| Tertiary degree | 13427 (49%) |
| Townsend Deprivation Index | -2.6 (IQR 3.3) |
| Self-reported ethnicity |  |
| British | 24793 (91%) |
| Other white | 796 (3%) |
| Irish | 685 (2.5%) |
| Indian | 180 (0.7%) |
| Other ethnic group | 136 (0.5%) |
| Hypertension | 8684 (32%) |
| Diabetes mellitus | 1288 (4.7%) |
| Hypercholesterolemia | 5550 (20.4%) |
| Stroke | 857 (3.2%) |
| Ever smoked | 9804 (36%) |
| Ever drunk alcohol | 26172 (96.4%) |
| Apolipoprotein E (*APO*ε4) carrier | 7321 (27%) |
| Systolic blood pressure (mmHg) | 139 ± 19 |
| Diastolic blood pressure (mmHg) | 78 ± 11 |
| Body mass index (kg/m^2^) | 26.3 ± 4.3 |
| Cardiac index (L/min/ m^2^) | 2.5 ± 0.8 |
| Total brain volume (mL) | 1,499 ± 73 |
| Gray matter volume (mL) | 795 ± 47 |
| White matter volume (mL) | 702 ± 41 |
| Volume of white matter hyperintensity (mL) | 4.6 ± 6.1 |
| Total hippocampal volume (mL) | 9.9 ± 1.1 |

**Supplementary Table 5** Associations and interactions between cardiac index (CI), age and sex on brain volumes prior to the exclusion of those with outlier values in LVEDV, LVESV and SV.

|  | **Model 1**  β  (95%CI)  p-value | **Model 2**  β  (95%CI)  p-value | **Model 3**  β  (95%CI)  p-value | **Model 4**  β  (95%CI)  p-value | **Model 5**  β  (95%CI)  p-value |
| --- | --- | --- | --- | --- | --- |
| **Total brain volume** (mL) | | | | | |
| CI | 5.59  (4.53 to 6.66)  <2×10^-16^ | 0.31  (-0.57 to 1.19)  0.49 | 0.09  (-1.01 to 1.19)  0.87 | -9.26  (-17.50 to -1.01)  0.03 | -20.44  (-34.97 to -5.91)  0.01 |
| Age × Sex |  | 0.83  (0.64 to 1.02)  <2×10^-16^ | 0.84  (0.65 to 1.02)  <2×10^-16^ | 0.82  (0.63 to 1.01)  <2×10^-16^ | 0.80  (0.57 to 1.02)  4.46×10^-12^ |
| CI × Sex |  |  | 0.62  (-1.21 to 2.45)  0.51 |  |  |
| CI × Age |  |  |  | 0.15  (0.02 to 0.28)  0.02 | 0.36  (0.12 to 0.57)  0.003 |
| **Gray matter volume** (mL) | | | | | |
| CI | 2.68  (1.99 to 3.38)  3.97×10^-14^ | -0.54  (-1.07 to -0.01)  0.045 | -0.68  (-1.34 to -0.01)  0.045 | -5.59  (-10.54 to -0.64)  0.03 | -5.28  (-10.23 to -0.32)  0.04 |
| Age × Sex |  | 0.53  (0.42 to 0.64)  <2×10^-16^ | 0.53  (0.42 to 0.65)  <2×10^-16^ | 0.52  (0.41 to 0.64)  <2×10^-16^ | 0.55  (0.44 to 0.67)  <2×10^-16^ |
| CI × Sex |  |  | 0.37  (-0.72 to 1.47)  0.50 |  |  |
| CI × Age |  |  |  | 0.08  (0.002 to 0.16)  0.04 | 0.06  (-0.007 to 0.15)  0.07 |
| **White matter volume** (mL) | | | | | |
| CI | 2.91  (2.31 to 3.51)  <2×10^-16^ | 0.85  (0.28 to 1.42)  0.003 | 0.77  (0.05 to 1.48)  0.04 | -3.68  (-9.04 to 1.68)  0.18 | -3.83  (-9.27 to 1.60)  0.17 |
| Age × Sex |  | 0.30  (0.18 to 0.42)  1.37×10^-6^ | 0.30  (0.18 to 0.43)  1.29×10^-6^ | 0.29  (0.17 to 0.42)  2.01×10^-6^ | 0.32  (0.18 to 0.44)  9.0×10^-7^ |
| CI × Sex |  |  | 0.24  (-0.95 to 1.43)  0.69 |  |  |
| CI × Age |  |  |  | 0.07  (-0.01 to 0.15)  0.09 | 0.07  (-0.01 to 0.16)  0.09 |
| **White matter hyperintensity volume** (mL) | | | | | |
| CI | -0.05  (-0.07 to -0.04)  1.16×10^-12^ | 0.013  (0.0002 to 0.025)  0.04 | 0.012  (-0.003 to 0.028)  0.11 | 0.093  (-0.03 to 0.21)  0.127 | 0.08  (-0.03 to 0.20)  0.16 |
| Age × Sex |  | -0.010  (-0.012 to -0.007)  8.85×10^-13^ | -0.010  (-0.01 to -0.007)  1.38×10^-12^ | -0.010  (-0.01 to -0.007)  1.40×10^-12^ | -0.009  (-0.01 to -0.006)  1.37×10^-10^ |
| CI × Sex |  |  | 0.001  (-0.03 to 0.027)  0.97 |  |  |
| CI × Age |  |  |  | -0.001  (-0.003 to 0.0006)  0.187 | -0.001  (-0.003 to 0.0005)  0.18 |
| **Total hippocampal volume** (mL) | | | | | |
| CI | 0.02  (0.01 to 0.04)  0.005 | -0.01  (-0.02 to 0.01)  0.37 | -0.01  (-0.03 to 0.01)  0.15 | -0.28  (-0.43 to -0.13)  2.0 ×10^-4^ | -0.29  (-0.44 to -0.14)  1.8×10^-4^ |
| Age × Sex |  | -0.01  (-0.02 to -0.01)  1.5×10^-14^ | -0.01  (-0.02 to -0.01)  7.6×10^-14^ | -0.01  (-0.02 to -0.01)  4.6×10^-15^ | -0.01  (-0.02 to -0.01)  3.69×10^-13^ |
| CI × Sex |  |  | 0.02  (-0.01 to 0.05)  0.23 |  |  |
| CI × Age |  |  |  | 0.004  (0.002 to 0.007)  2.7×10^-4^ | 0.004  (0.002 to 0.007)  2.71×10^-4^ |

**Key:** Model 1: univariable; Model 2: Model 1 + age × sex interaction term; Model 3: Model 2 + CI × sex interaction term; Model 4: Model 2 + CI × age interaction term; Model 5 Model 4 + ethnicity, ApoE ε4 status, Townsend Deprivation Index, systolic blood pressure, diastolic blood pressure, diabetes, hypertension, stroke, hypercholesterolemia, Body Mass Index, alcohol and smoking status
